# Annual Meeting of the American Medical Association

**Published:** 1856-02

**Authors:** 


*Annual Meeting of the American Medical Association.*

The next Annual Meeting of the Association is to be held in Detroit, commencing on the first Tuesday in May. We trust this will be borne in mind, more especially by the physicians of the North-west. Every regular organized state, county, or district medical society is entitled to send one delegate to every 10 of its members. If there are any societies in this or the adjoining states, whose delegates are not already selected, such selection should be made as early as possible, and the names of the delegates forwarded to Dr. WM. BODIE, of Detroit, one of the secretaries of the association. The approaching annual meeting will doubtless be one of as much interest and importance as any that have preceded it, in its scientific and business relations. But whether the NUMBER in attendance shall be equal to former occasions, will depend very much on the profession of Michigan and the states immediately surrounding her. We trust, therefore, tha ta just local pride or emulation will stir up our professional brethren, throughout the North-west, and cause them to extend their social organizations, and see to it that such delegates are appointed as will attend the approaching meeting.

